# Sex Differences in the Rapid Cell Signaling Mechanisms Underlying the Memory-Enhancing Effects of 17β-Estradiol

**DOI:** 10.1523/ENEURO.0267-18.2018

**Published:** 2018-10-30

**Authors:** Wendy A. Koss, Jacqueline M. Haertel, Sarah M. Philippi, Karyn M. Frick

**Affiliations:** 1 Department of Psychology, University of Wisconsin-Milwaukee, Milwaukee, WI 53211

**Keywords:** ERK, hippocampus, memory consolidation, object recognition, spatial memory

## Abstract

Little is known about how 17β-estradiol (E_2_) mediates memory formation in males. In ovariectomized (OVX) mice, bilateral dorsal hippocampal (DH) infusion of E_2_ enhances memory consolidation in object recognition (OR) and object placement (OP) tasks in a manner dependent on activation of extracellular signal-regulated kinase (ERK) and Akt signaling. Here, bilateral DH E_2_ infusion enhanced memory consolidation in both tasks among OVX female, gonadally-intact male, and castrated male mice, suggesting comparable facilitation of memory consolidation in both sexes, independent of testicular hormones in males. Contrary to previous reports in OVX mice, E_2_ did not increase DH ERK or Akt phosphorylation in males, nor did the ERK inhibitor U0126 [1,4-diamino-2,3-dicyano-1,4-bis (o-aminophenylmercapto) butadiene] prevent E_2_ from enhancing memory consolidation among intact and castrated males. These data suggest that ERK activation is not necessary for E_2_ to enhance memory consolidation in males, and compared with previous reports in females, reveal novel sex differences in the cell-signaling pathways through which E_2_ facilitates memory consolidation. To explore the mechanisms underlying E_2_-induced memory enhancements in males, phosphorylation of the transcription factor cAMP response element binding protein (CREB) in the DH was assessed. E_2_ increased phospho-CREB levels in both sexes, yet U0126 did not block these increases in castrated or intact males, indicating that E_2_ regulates CREB phosphorylation in males via an ERK-independent mechanism. Collectively, these findings suggest that the beneficial effects of hippocampal E_2_ on memory consolidation in males and females are mediated by different molecular mechanisms, which has important implications for the development of treatments to reduce memory dysfunction in men and women.

## Visual Abstract

**Figure:**
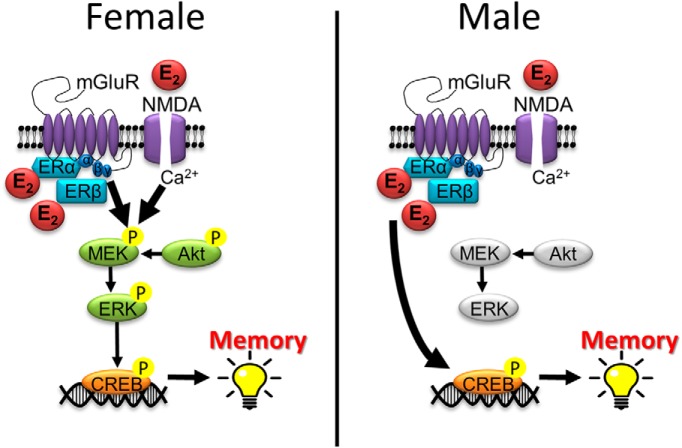

## Significance Statement

The impact of biological sex on neural function is understudied, despite clear sex differences in the risks and symptomology of disorders such as Alzheimer’s disease, mood disorders, developmental learning disabilities, and attention-deficit disorder. The ability of 17β-estradiol (E_2_) to enhance memory consolidation in females depends on rapid activation of cell signaling pathways in the dorsal hippocampus (DH), yet the mechanisms underlying its effects in males are unknown. The present study suggests a novel sex difference in the hippocampal cell-signaling mechanisms through which E_2_ facilitates memory consolidation. These and future studies may lead to the development of sex-specific therapeutics that more effectively reduce cognitive dysfunction in diseases that disproportionately affect one sex or the other.

## Introduction

Interest in sex differences is on the rise, perhaps due to the National Institutes of Health mandate that sex be considered as a biological variable in research proposals. Unlike most areas of neuroscience, in which male subjects predominate, females have typically been the focus of studies examining hormonal regulation of cognition. Ample evidence demonstrates that 17β-estradiol (E_2_) given to ovariectomized (OVX) rat and mice enhances object recognition (OR) and spatial memory consolidation (Tuscher et al., 2015; Sheppard et al., 2018). Numerous receptor, cell-signaling, and epigenetic mechanisms necessary for these effects have been identified (Frick, 2015; Frick et al., 2015, 2018; Galea et al., 2017). In contrast, the effects of E_2_ on memory processes in male rodents have not been well studied, and the mechanisms underlying these effects are scarcely known.

Studies using various spatial tasks have demonstrated that pre-training E_2_ administration improves spatial learning and memory among intact and gonadectomized (GDX) male rodents (Luine and Rodriguez, 1994; Heikkinen et al., 2002; Gibbs, 2005; Gibbs and Johnson, 2008; McConnell et al., 2012; Locklear and Kritzer, 2014). In studies using acute post-training treatments to pinpoint effects of E_2_ on spatial memory consolidation, systemic administration enhanced consolidation in an object placement (OP) task among GDX male rats (Jacome et al., 2016), and dorsal hippocampal (DH) infusion enhanced consolidation in the Morris water maze among intact male rats (Packard et al., 1996). Although evidence is limited, these few studies suggest that E_2_ facilitates memory consolidation in males as it does in females. However, unlike females, the mechanisms through which E_2_ enhances memory in males are unclear.

The male hippocampus expresses all three major estrogen receptors (ERα, ERβ, and GPER; Milner et al., 2001, 2005; Mitterling et al., 2010; Waters et al., 2011, 2015), but sex differences in ER distribution are observed within dendritic spines (Gillies and McArthur, 2010). In OVX rats and mice, acute administration of E_2_ systemically or into the DH rapidly increases CA1 dendritic spine density, an effect that depends on phosphorylation of extracelluar signal-regulated kinase (ERK) and downstream mammalian target of rapamycin (mTOR) signaling (Inagaki et al., 2010; Tuscher et al., 2016a). Similarly, activation of ERK and other signaling pathways is necessary for E_2_ to increase CA1 spine density in adult male hippocampal slices (Murakami et al., 2006; Mukai et al., 2007). Because E_2_ enhances memory consolidation in both sexes, and ERK phosphorylation is necessary for E_2_-induced memory consolidation in OVX females (Boulware et al., 2013), ERK activation could be involved in the mnemonic effects of E_2_ in males. However, E_2_ administered to neonatal rat hippocampal cultures induces ERK-dependent phosphorylation of the transcription factor cAMP response element binding protein (CREB) in female-derived cultures, but not cultured tissue from males (Boulware et al., 2005), suggesting potential sex differences in E_2_-induced ERK phosphorylation. As such, the extent to which estrogenic regulation of memory in males might depend on ERK activation is unclear.

Given the many gaps in the male literature, this study was designed to determine whether DH E_2_ infusion affects memory consolidation and DH ERK phosphorylation in adult male mice. We first compared effects of post-training E_2_ on object recognition and spatial memory consolidation in intact male and OVX female mice. E_2_ enhanced consolidation of both types of memory in both sexes, but only increased phosphorylation of p42 ERK and upstream Akt in females. Moreover, inhibiting ERK phosphorylation did not block the memory-enhancing effects of E_2_ in males, suggesting that E_2_-induced memory enhancement in intact males does not depend on DH ERK activation. To determine whether testicular hormones affect this sex difference, we compared effects of E_2_ on memory consolidation and phosphorylation of ERK and Akt in GDX and intact males. As before, E_2_ enhanced memory consolidation without affecting ERK or Akt, and ERK inhibition did not block E_2_’s mnemonic effects. However, E_2_ increased CREB phosphorylation in both sexes, and this effect was not blocked by ERK inhibition in males, suggesting that E_2_ may regulate memory in males via ERK-independent CREB phosphorylation.

## Materials and Methods

### Subjects

Female and male mice were obtained from Taconic Biosciences at nine weeks of age. Mice were maintained on a 12/12 h light/dark cycle with *ad libitum* access to food and water. Mice underwent surgeries at 10 weeks of age and behavioral testing began at ∼11–15 weeks of age. Mice were group-housed until surgery, after which point they were single housed. All procedures followed the National Institutes of Health Guidelines for the Care and Use of Laboratory Animals and were approved by the Institutional Animal Care and Use Committee of the University of Wisconsin-Milwaukee.

### Surgeries

Mice were administered 5% isoflurane in 100% oxygen for anesthesia induction and then maintained at 2–3% isoflurane throughout surgery. All mice underwent a two-step surgical procedure in which they were first OVX, GDX, or sham GDX and were then implanted intracranially with chronic indwelling guide cannulae. All females were OVX as described previously (Boulware et al., 2013; Kim et al., 2016; Tuscher et al., 2016a). Briefly, bilateral incisions were made on each side of the lower dorsal flanks, followed by an incision in each underlying muscle wall. Each side of the fallopian tubes was ligated and ovaries were removed. Muscle wall incisions were sutured and skin incisions were closed with wound clips. In experiments where males were compared to females, males were left gonadally-intact but underwent similar anesthesia and bilateral incisions as females. In experiments comparing gonadally-intact (sham) males to GDX males, intact males underwent similar anesthetic and procedures as GDX males. During male GDX surgeries, a midline incision was made on the scrotal sac, testes were isolated and carefully separated from fat, and then the testes were tied off at the vas deferens and spermatic artery with chromic gut and the testes were removed. The incision was closed with monofilament sutures.

Immediately following sham and GDX procedures, all mice were implanted with chronic indwelling guide cannulae as described previously (Boulware et al., 2013; Fortress et al., 2013; Kim et al., 2016). Mice were secured in a stereotaxic apparatus (Kopf Instruments) and implanted with bilateral guide cannulae (22 gauge; C232G, Plastics One Inc.) aimed at each hemisphere of the DH (−1.7 mm AP, ±1.5 mm ML, −2.3 mm DV) or with triple guide cannulae aimed at both hippocampi and the dorsal third ventricle (intracerebroventricular; −0.9 mm AP, ±0.0 mm ML, −2.3 mm DV). Dummy cannulae (C232DC, Plastics One Inc.) were inserted into all guide cannulae to maintain patency. Cannulae were fixed to the skull with dental cement (Darby Dental Supply) that served to close the wound.

### Drugs and infusions

Infusions were performed by gently restraining mice, removing the dummy cannulae, and placing an infusion cannula into the guide cannulae (C313I; DH: 28 gauge, extending 0.8 mm beyond the 1.5 mm guide; intracerebroventricular, 28 gauge, extending 1.0 mm beyond the 1.8 mm guide). The infusion cannula was attached to PE50 polyethylene tubing that was mounted on a 10-μl Hamilton syringe. Infusions were controlled by a microinfusion pump (KDS Legato 180, KD Scientific) and conducted immediately post-training at a rate of 0.5 μl/min into the DH or 1 μl/2 min into the dorsal third ventricle as described previously (Boulware et al., 2013; Fortress et al., 2013, 2014; Kim et al., 2016). Infusion cannulae remained in place for 1 min after each infusion to prevent diffusion back up the cannula track. For studies in which E_2_ was infused in combination with the ERK phosphorylation inhibitor U0126 [1,4-diamino-2,3-dicyano-1,4-bis (o-aminophenylmercapto) butadiene], the inhibitor was first infused bilaterally into the DH and then E_2_ was infused to the dorsal third ventricle. This triple infusion protocol prevents possible damage to the DH from two infusions in rapid succession (Fernandez et al., 2008; Zhao et al., 2010, 2012; Boulware et al., 2013; Fortress et al., 2013).

Cyclodextrin-encapsulated E_2_ (Sigma-Aldrich Corp.) was dissolved in 0.9% saline and infused at doses of 5 μg/hemisphere into the DH or 10 μg into the dorsal third ventricle. The vehicle, 2-hydroxypropyl-β-cyclodextrin (HBC; Sigma- Aldrich Corp.) dissolved in 0.9% saline, was used at the same concentration as encapsulated E_2_ (Zhao et al., 2012; Boulware et al., 2013). DH or intracerebroventricular infusion of these E_2_ doses enhances both OR and OP memory consolidation in OVX mice (Zhao et al., 2010, 2012; Boulware et al., 2013; Fortress et al., 2013; Kim et al., 2016). The ERK phosphorylation inhibitor U0126 (Promega Corp.) was dissolved in 50% DMSO and infused at doses of 0.25, 0.5, or 1 μg/hemisphere into the DH. The vehicle control for U0126 was 50% DMSO in 0.9% saline. In OVX mice, bilateral DH infusion of 1 μg, but not 0.1 or 0.5 µg, U0126 impairs OR memory consolidation (Fernandez et al., 2008), and DH infusion of 0.5 µg does not impair OP memory consolidation (Boulware et al., 2013). Because 0.5 µg/hemisphere U0126 has no detrimental effects on memory consolidation tested in either task, we previously infused this dose into the DH of OVX mice in conjunction with an intracerebroventricular infusion of 10 µg E_2_ and found that U0126 blocked the memory-enhancing effects of E_2_ in both tasks (Fernandez et al., 2008; Fortress et al., 2013). Here, we used these data as a guide for experiments in intact and GDX males to identify a behaviorally subeffective dose of U0126 that could be used to determine whether the memory-enhancing effects of E_2_ in males depend on ERK activation.

### OP and OR tasks

Approximately one week after surgery, mice began OP and OR testing to assess spatial and OR memory consolidation, respectively, as described previously (Fernandez et al., 2008; Fan et al., 2010; Zhao et al., 2010, 2012; Boulware et al., 2013; Fortress et al., 2013). Memory in both tasks depends on intact function of the DH (Baker and Kim, 2002; Cohen et al., 2013) and administration of E_2_ immediately, but not 3 h, after training enhances memory consolidation in both tasks among young adult and middle-aged OVX mice (for review, see Tuscher et al., 2015). As in previous work (Tuscher et al., 2015), all treatments in this study were administered immediately after training to pinpoint effects on the consolidation phase of memory formation.

All mice were tested in both the OP and OR tasks, the order of which was counterbalanced within each group. OP testing, OR testing, and brain tissue collection were separated by two weeks to allow acute effects of each infusion to dissipate before the next infusion. Before the start of behavioral testing, mice were handled for 3 d and then habituated to the testing arena (60 × 60 × 47 cm high) for 5 min/d for 2 d. During habituation, mice moved about freely in the apparatus without objects present. During both handling and habituation, mice were acclimated to objects by placing a Lego Duplo brick (6.3 × 3.1 × 2.3 cm) in their home cage. Following habituation, mice were trained in either OP or OR. Mice were first briefly rehabituated to the arena by placing them inside without any objects for 2 min, after which they were returned to their home cage while two identical objects were placed in the northwest or northeast corners of the arena. Mice were then returned to the arena and allowed to explore the objects, which included a plumbing valve, chip clip, mini-stapler, date stamp, master lock, and aquarium figurines. To ensure that all mice received the same amount of exposure to the objects, trials were not ended until mice had accumulated 30 s of object exploration or after a maximum of 20 min. Object exploration was scored only when the mouse’s nose or front paws contacted an object. Immediately after the completion of the training session, mice were infused and then returned to their home cages.

Testing occurred 24 h later for OP and 48 h later for OR. During OP testing, one training object was moved to the southwest or southeast corner of the arena. During OR testing, one training object was replaced with a novel object. In both tasks, mice were required to explore the objects until they accumulated of 30 s of exploration. Time spent with the objects was recorded using ANY-maze tracking software (ANY-maze, RRID:SCR_014289). Mice that remember the identity and location of the training objects should spend more time than chance with the moved and novel objects. Chance is designated as 15 s because this value represents equal exploration of both objects, and hence, no memory of the training objects. The 24- and 48-h delays were used for OP and OR, respectively, based on previous evidence from OVX females that vehicle-treated mice do not show intact memory at 24 and 48 h after OP and OR training, respectively (Boulware et al., 2013; Fortress et al., 2015; Kim et al., 2016). Similarly, gonadally-intact males can remember object identity 24 h after OR training (Frick and Gresack, 2003; Fortress et al., 2013). Thus, the 24- and 48-h delays were used for OP and OR, respectively, to permit observation of potential memory-enhancing effects of E_2_. To test for impairing effects of U0126, delays were set at 4 h for the OP task and 24 h for OR. These delays were chosen because vehicle-treated OVX mice exhibit intact memory using these delays between training and testing (Boulware et al., 2013; Fortress et al., 2015; Kim et al., 2016).

### Western blotting

Two weeks after the conclusion of behavioral testing, mice were infused with vehicle or E_2_ and then cervically dislocated and decapitated 5 min later. The DH was then bilaterally dissected by carefully removing the overlying cortex and cutting the hippocampus horizontally at the level of the base of the superior colliculus. After severing the fornix, DH tissue was excised using forceps and stored at –80°C until homogenization. Western blotting was then performed as described previously by our laboratory (Boulware et al., 2013; Fortress et al., 2015; Kim et al., 2016). Tissue was homogenized in lysis buffer containing PMSF and a protease inhibitor cocktail (Sigma-Aldrich Corp.) using a sonicator (Sonifier 250, Branson Ultrasonics). Protein concentrations were measured using a Bradford assay (Bio-Rad Laboratories) and aliquots were prepared containing 2 µg/µl of protein. Proteins were electrophoresed on 10% TGX stain-free precast gels (Bio-Rad Laboratories) and transferred to PVDF membranes (Bio-Rad Laboratories). To verify protein transfer, total protein in each lane was visualized on a ChemiDoc MP gel imager (Bio-Rad Laboratories). Membranes were then blocked in 5% milk in 0.1% Tween 20 in Tris-buffered saline (TTBS) and incubated in primary antibodies overnight at 4°C. All primary antibodies were purchased from Cell Signaling Technologies (phospho-ERK #9101S, RRID:AB_331646 and total-ERK #9102S, RRID:AB_330744, 1:2000; phospho-AKT #9271S, RRID:AB_329825 and total AKT #4691S, RRID:AB_915783, 1:1000; phospho-CREB #9198S, RRID:AB_2561044, 1:750) and diluted in 5% BSA/TTBS. Blots were then incubated for 1 h at room temperature with a rabbit HRP-conjugated secondary antibody (Cell Signaling Technologies #7074S, RRID:AB_2099233; 1:15000 for ERK antibodies; 1:5000 for CREB and AKT) and developed using Clarity Max chemiluminescent substrate (Bio-Rad Laboratories). The ChemiDoc MP imager was used to detect the chemiluminescent signal. Densitometry was performed using Image Lab software (Bio-Rad Laboratories Image Lab v 5.2, RRID:SCR_014210). Both ERK (p44 and p42 isoforms) and Akt were normalized to their respective total protein and expressed as % immunoreactivity to vehicle. Phospho-CREB was normalized to total amount of protein transferred in each corresponding lane and expressed in % immunoreactivity to vehicle of the same sex. In experiments using U0126, % immunoreactivity was expressed relative to the group that received vehicle infusions into both the DH and dorsal third ventricle (vehicle/vehicle group).

### Experimental design and statistical analysis

#### Experiment 1

This experiment examined effects of E_2_ on memory consolidation in OVX female and sham GDX male mice. A 2 × 2 design was used with sex and treatment (vehicle vs E_2_) as between-subjects variables tested in a two-way ANOVA. One-sample *t* tests were also performed between each group mean and the hypothetical mean of 15 s (chance performance) to assess learning within each group. Female mice were OVX and males were sham GDX immediately before being implanted with bilateral DH guide cannulae. Immediately after training in OR and OP, mice received DH infusion of vehicle (OR: male, *n* = 20, female, *n* = 18; OP: male, *n* = 22, female, *n* = 18) or E_2_ (OR: male, *n* = 22, female, *n* = 18; OP: male, *n* = 20, female, *n* = 17) and were tested as described above. Two weeks after the completion of behavioral testing, the intact males and OVX females received bilateral DH infusions of vehicle (male, *n* = 14; female, *n* = 14) or E_2_ (male, *n* = 17; female, *n* = 18) and DH tissue was collected 5 min later for Western blot analysis of ERK phosphorylation. A subset of these brains (*n* = 6/group) was also assayed for Akt and CREB phosphorylation.

#### Experiment 2

This study tested effects of E_2_ on memory consolidation in sham GDX and GDX male mice. A 2 × 2 design was used, with surgery (sham or GDX) and treatment (vehicle vs E_2_) as between-subjects variables tested in a two-way ANOVA. One-sample *t* tests were also performed to assess within-group learning. Males received sham GDX or GDX surgery immediately before bilateral cannula implanation surgery. Mice received vehicle (OR: sham, *n* = 10, GDX, *n* = 9; OP: sham, *n* = 10, GDX, *n* = 10) or E_2_ (OR: sham, *n* = 9, GDX, *n* = 8; OP: sham, *n* = 9, GDX, *n* = 11) infusion immediately after OR and OP training and were tested as described above.

#### Experiment 3

Vehicle or one of three doses of the ERK phosphorylation inhibitor U0216 were infused into the DH to identify a dose that would not impair memory on its own in a subsequent co-infusion experiment with E_2_. U0126 prevents the upstream kinase MAP kinase (MAPK) kinase (MEK) from phosphorylating ERK. Male mice were GDX and implanted with bilateral DH cannulae. Immediately after OR and OP training, mice received DH infusion of 50% DMSO vehicle (OR: *n* = 10; OP: *n* = 10) or U0126 in doses of 0.25 μg/hemisphere (OR: *n* = 9; OP: *n* = 6), 0.5 μg/hemisphere (OR: *n* = 8; OP: *n* = 8), or 1 μg/hemisphere (OR: *n* = 7; OP: *n* = 8). A one-way ANOVA was performed to assess differences among the groups, and one-sample *t* tests assessed within-group learning.

#### Experiments 4a and 4b

In experiment 3, 0.5 μg/hemisphere U0126 was found to have no effect on memory in either behavioral task, and so was used here to determine whether ERK phosphorylation is necessary for the memory-enhancing effects of E_2_ in sham GDX (experiment 4a) and GDX males (experiment 4b). Both studies used 2 × 2 designs in which hormone treatment (vehicle or E_2_) and inhibitor treatment (vehicle or U0126) were the between-subjects variables in two-way ANOVAs. Again, one-sample *t* tests assessed learning within each group. Immediately after training, mice were infused with vehicle or 0.5 µg/hemisphere U0126 into the DH and vehicle or 10 µg E_2_ into the dorsal third ventricle. In experiment 4a, samples sizes for sham GDX mice were as follows for OR (vehicle+vehicle: *n* = 8; vehicle+U0126: *n* = 8; E_2_+vehicle: *n* = 9; E_2_+U0126: *n* = 10) and OP (vehicle+vehicle: *n* = 5; vehicle+U0126: *n* = 5; E_2_+vehicle: *n* = 7; E_2_+U0126: *n* = 7). In experiment 4b, samples sizes for GDX mice were as follows for OR (vehicle+vehicle: *n* = 13; vehicle+U0126: *n* = 6; E_2_ + vehicle: *n* = 11; E_2_+U0126: *n* = 9) and OP (vehicle + vehicle: *n* = 7; vehicle + U0126: *n* = 7; E_2_ + vehicle: *n* = 8; E_2_ + U0126: *n* = 9). Testing was conducted as described above. Two weeks later, mice were infused again and the DH dissected for Western blot analysis of ERK (*n* = 6/group), Akt (*n* = 6/group), and CREB levels (*n* = 5-6/group).

### Statistical analyses

For all behavioral experiments, two statistical analyses were performed using GraphPad Prism 6 software (RRID:SCR_002798; Kim et al., 2016; Tuscher et al., 2016b; Kim and Frick, 2017). To assess learning within each group, one-sample *t* tests compared exploration time for each group with chance (set at 50% or 15 s). More time spent with the moved or novel object indicated intact memory for the location and identity, respectively, of the training objects. To assess effects of sex and treatment among the groups, one- or two-way ANOVAs were performed, followed by Fisher’s LSD *post hoc* tests for significant interactions when necessary. Similar ANOVA and *post hoc* analyses were conducted for Western blotting data. Significance was defined at *p* < 0.05.

## Results

### E_2_ enhances memory consolidation in gonadally-intact males and OVX females

**Figure 1.**
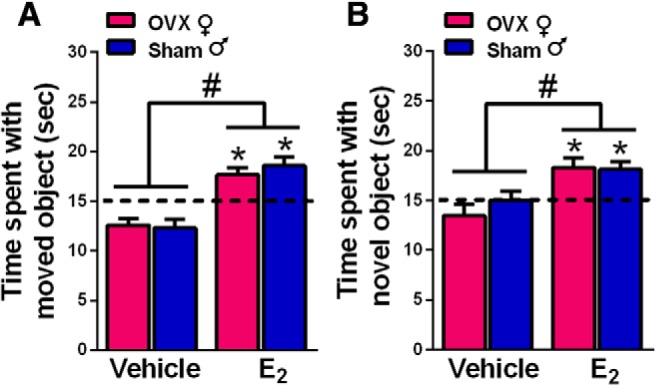
Infusion of E_2_ into the DH enhanced spatial and OR memory consolidation in both sexes in the OP (***A***) and OR (***B***) tasks. OVX female and sham GDX male mice were infused with E_2_ immediately after OP and OR training. Both female and male mice treated with E_2_ investigated the moved (***A***) or novel (***B***) object more than chance (*p < 0.05; chance = dashed line at 15 s). E_2_-treated groups also interacted with the moved or novel object significantly more than same-sex vehicle-treated mice (#*p* < 0.05). Each bar represents the mean +/− standard error of the mean (SEM).

In OVX females, DH infusion of E_2_ consistently enhances 24-h OP and 48-h OR memory consolidation (Tuscher et al., 2015; Frick et al., 2018). To explore potential sex differences in the memory-enhancing effects of DH E_2_ infusion, OVX females and sham GDX males received bilateral DH infusion of vehicle or 5 µg/hemisphere E_2_ immediately after training in both object tasks (experiment 1). Two-way ANOVAs revealed significant main effects of E_2_ treatment for both OP (*F*_(1,69)_ = 48.79, *p* < 0.0001; Fig. 1*A*) and OR (*F*_(1,73)_ = 18.27, *p* < 0.0001; Fig. 1*B*), indicating that vehicle groups differed significantly from E_2_-treated groups in both tasks. The main effect of sex and sex × treatment interaction were not significant, suggesting similar memory-enhancing effects of E_2_ in both sexes. These data are supported by the results of one-sample *t* tests, which assess whether the time each group spent with the moved or novel object differs relative to the chance value of 15 s. In OP, E_2_-treated groups of both sexes spent significantly more time exploring the displaced object than chance (male, *t*_(18)_ = 4.16, *p* = 0.003; female, *t*_(15)_ = 3.63, *p* = 0.003), whereas vehicle-infused groups spent significantly less time than chance exploring the moved object (male, *t*_(19)_ = 3.06, *p* = 0.006; female, *t*_(17)_ = 3.44, *p* = 0.003). In OR, E_2_-treated mice of both sexes interacted with the novel object significantly more than chance (male, *t*_(21)_ = 3.88, *p* = 0.0009; female, *t*_(16)_ = 3.44, *p* = 0.003), whereas vehicle-infused groups did not perform significantly different from chance. These data illustrate that DH infusion of E_2_ enhances OR and spatial memory consolidation in both OVX females and gonadally-intact males.

### E_2_ enhances memory consolidation in gonadally-intact and GDX males

**Figure 2.**
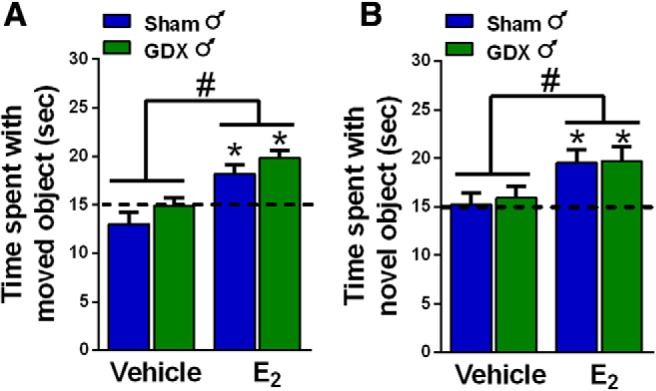
Infusion of E_2_ into the DH enhanced spatial and OR memory consolidation in both intact and GDX males. E_2_-treated mice explored the moved (***A***) or novel (***B***) object in the OP and OR tasks significantly more than chance (*p < 0.05). Both E_2_-treated male groups also spent significantly more time with the moved or novel object than vehicle-treated controls (#*p* < 0.05). Each bar represents the mean +/− SEM.

In males, the testes are the primary source of circulating androgens and estrogens, where estrogens are synthesized from testosterone by the enzyme aromatase. Both testosterone and E_2_ regulate memory in males (Luine and Rodriguez, 1994; Vázquez-Pereyra et al., 1995; Packard et al., 1996; Gibbs, 2005; Kritzer et al., 2007; McConnell et al., 2012), so the presence of the testes in our study of gonadally-intact males may have influenced our findings in experiment 1. To address this possibility, sham GDX and GDX males were infused with vehicle or E_2_ into the DH immediately after training in OP and OR (experiment 2). Two-way ANOVAs revealed significant main effects of E_2_ treatment for OP (*F*_(1,35)_ = 22.84, *p* < 0.0001; Fig. 2*A*) and OR (*F*_(1,32)_ = 9.72, *p* = 0.0038; Fig. 2*B*). These data suggest that the loss of the testes does not prevent DH E_2_ from enhancing memory, and indicates that E_2_ can enhance memory in males regardless of the presence o*f* testicular hormones. One-sample *t* tests confirmed that both E_2_-treated groups spent significantly more time with the moved and novel objects than chance (sham+E_2_, OP: *t*_(7)_ = 3.43, *p* = 0.01, OR: *t*_(8)_ = 3.37, *p* = 0.01; GDX+E_2_, OP: *t*_(10)_ = 6.15, *p* = 0.0001, OR: *t*_(7)_ = 3.28, *p* = 0.01), whereas the vehicle-treated groups did not. Together, these analyses demonstrate that E_2_ enhances OR and spatial memory consolidation similarly in gonadally-intact and GDX male mice.

### E_2_ does not increase DH ERK or Akt phosphorylation in gonadally-intact males

**Figure 3.**
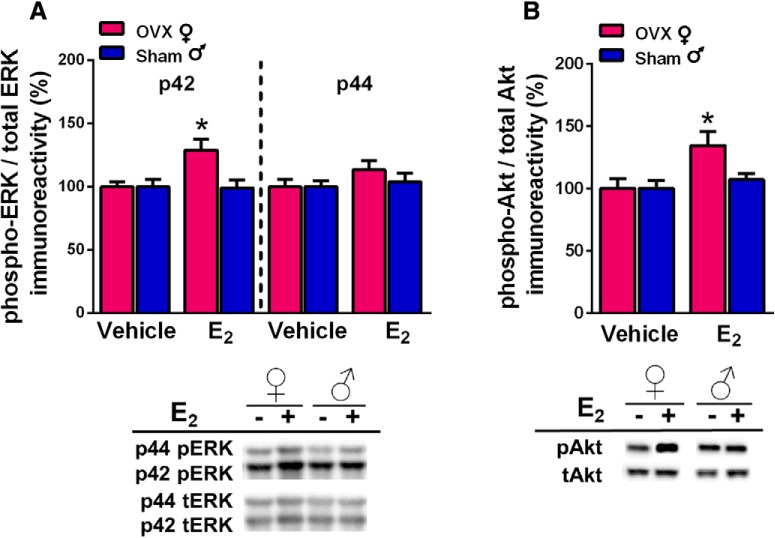
DH E_2_ infusion significantly increased phosphorylation of the p42 isoform of ERK (***A***) and of Akt (***B***) in the DH of OVX females, but not sham GDX males. ***A***, Levels of phospho-p42, but not phospho-p44, ERK were significantly increased by E_2_ in OVX females but not sham males. ***B***, E_2_ also significantly increased DH phospho-Akt levels in females but not males. Representative blots are shown below both graphs; *p < 0.05 relative to the same-sex vehicle-infused group. Each bar represents the mean +/− SEM.

DH E_2_ infusion significantly increases phosphorylation of the p42 isoform of ERK (p42-ERK) in the DH of OVX mice within 5 min (Fernandez et al., 2008; Fan et al., 2010; Boulware et al., 2013; Fortress et al., 2013; Kim et al., 2016). Moreover, ERK phosphorylation in the DH is required for E_2_ to enhance memory consolidation in OR and OP, as demonstrated by studies in which DH infusion of the ERK phosphorylation inhibitor U0126 prevented intracerebroventricularly-infused E_2_ from facilitating memory consolidation in OVX mice (Fernandez et al., 2008; Fortress et al., 2013). To determine whether similar underlying mechanisms are responsible for E_2_-induced memory consolidation in males, DH homogenates were obtained from the OVX females and sham GDX males in which E_2_ enhanced memory consolidation (experiment 1). Two weeks after the completion of behavioral testing, mice received bilateral DH infusions of vehicle or E_2_, and dorsal hippocampi were bilaterally dissected 5 min later. Phospho-p42 ERK levels were significantly increased in OVX females, but not in intact males (Fig. 3*A*), as shown by a significant sex × treatment interaction (*F*_(1,59)_ = 5.11, *p* = 0.028), as well as main effects of treatment (*F*_(1,59)_ = 4.57, *p* = 0.037) and sex (*F*_(1,59)_ = 5.11, *p* = 0.028). *Post hoc* analyses revealed that E_2_-treated females had elevated phospho-p42 ERK levels relative to vehicle-treated females (*p* = 0.027) and E_2_-treated males (*p* = 0.0013), whereas phosho-p42 ERK levels did not differ between the male groups. As in our previous studies with OVX females (Fernandez et al., 2008; Zhao et al., 2010; Boulware et al., 2013; Fortress et al., 2013; Kim et al., 2016), E_2_ had no significant effect on the phosphorylation of the p44 isoform of ERK in either sex.

Given that ERK phosphorylation was not increased in sham GDX males, we sought converging evidence from an upstream signaling pathway known to activate ERK. Akt is part of the phosphoinositol 3-kinase (PI3K)/Akt signaling pathway. Our previous work in OVX females showed that E_2_ increased phosphorylation of both PI3K and Akt within 5 min of DH infusion, and that activation of PI3K was necessary for E_2_ to increase both p42 ERK phosphorylation and OR memory consolidation (Fan et al., 2010; Fortress et al., 2013). Thus, we used phosphorylated Akt as an indicator of whether E_2_ activated the PI3K/Akt pathway in a subset of the OVX female and sham GDX male samples examined above. Two-way ANOVA indicated a significant main effect of E_2_ treatment (*F*_(1,39)_ = 14.4, *p* = 0.0096; Fig. 3*B*) and trends for a main effect of sex (*F*_(1,39)_ = 6.32, *p* = 0.079) and sex × treatment interaction (*F*_(1,39)_ = 6.32, *p* = 0.079). *Post hoc* tests revealed that E_2_-treated females had elevated phospho-Akt levels relative vehicle-treated females (*p* = 0.0044) and E_2_-treated males (*p* = 0.0162), whereas phospho-Akt levels did not differ significantly between the male groups. These data provide additional evidence that ERK and upstream signaling kinases in the DH are not necessary for E_2_ to enhance memory in sham GDX males.

### ERK phosphorylation is not necessary for E_2_ to enhance memory in GDX or gonadally-intact males

Although the lack of ERK and Akt activation in sham GDX males suggests that DH E_2_ infusion does not activate these pathways within 5 min, it remains possible that these signaling kinases are affected at later time points where they could facilitate memory consolidation. In OVX mice, our laboratory has shown that bilateral DH infusion of a behaviorally subeffective dose of the ERK phosphorylation inhibitor U0126 (0.5 µg/hemisphere) prevents intracerebroventricularly-infused E_2_ from enhancing OP and OR memory consolidation (Fernandez et al., 2008; Fortress et al., 2013). Here, the identical experiment was performed in GDX and sham GDX males to more definitively assess the role of ERK activation in the memory-enhancing effects of E_2_ in males.

**Figure 4.**
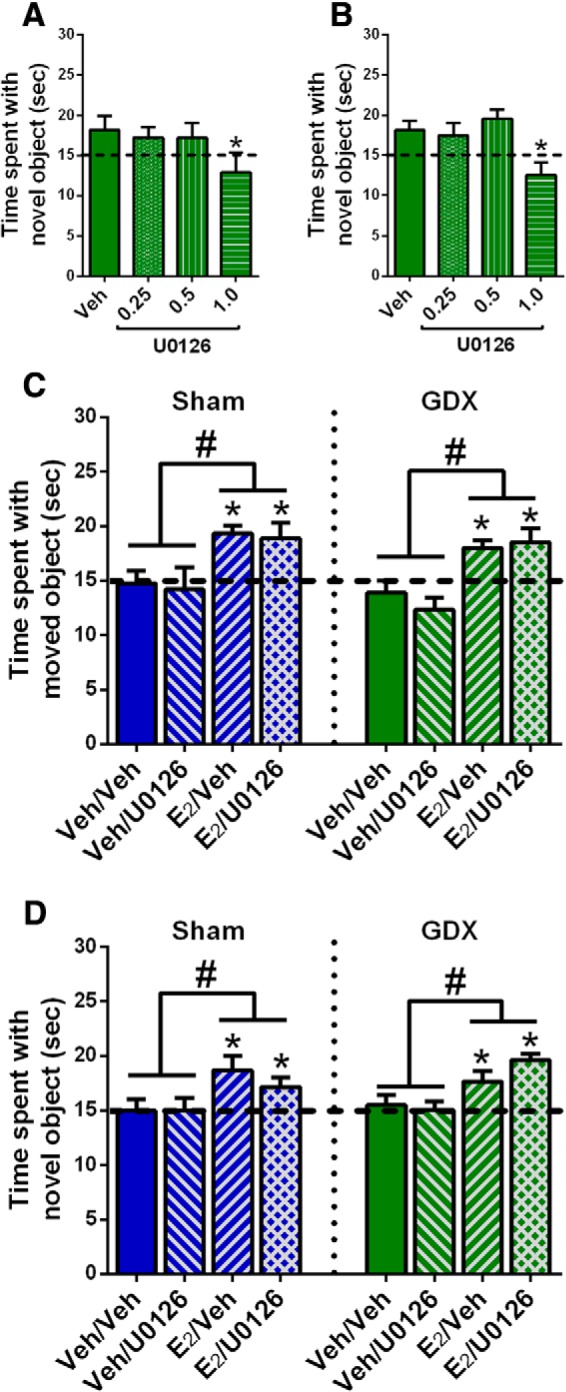
The ERK phosphorylation inhibitor U0126 did not block the memory-enhancing effects of E_2_ in intact or GDX males. ***A***, ***B***, Three doses of U0126 were tested to find one that did not impair memory consolidation on its own. In both OP (***A***) and OR (***B***), 1.0 μg/hemisphere U0126 impaired memory consolidation, whereas 0.5 μg/hemisphere U0126 did not. As such, 0.5 μg/hemisphere U0126 was next co-infused with E_2_ to determine whether the beneficial effects of E_2_ on memory in males depended on ERK phosphorylation. ***C***, ***D***, Sham and GDX male mice receiving intracerebroventricular infusion of E_2_ spent significantly more time than chance (*p < 0.05; dashed line) exploring the moved or novel object in OP (***C***) and OR (***D***), regardless of whether they received a DH infusion of vehicle (Veh) or U0126. In contrast, mice receiving intracerebroventricular vehicle and DH vehicle or U0126 did not differ from chance in time spent with the moved and novel objects. Time spent with the novel and moved objects in the E_2_-infused groups was significantly higher than that in the vehicle groups, independent of U0126 administration (#*p* < 0.05). Each bar represents the mean +/− SEM.

Before performing this study, it was first necessary to identify a dose of U0126 that did not affect memory on its own in males (experiment 3). This step was important to ensure that any memory-impairing effects of E_2_ and U0126 given in combination were not due to a general memory-impairing effect of U0126 (Fernandez et al., 2008). In OVX females, bilateral DH infusion of 1 µg/hemisphere U0126 impairs OP and OR memory consolidation, whereas 0.5 and 0.1 µg/hemisphere do not (Fernandez et al., 2008; Boulware et al., 2013). In the present study, GDX males were bilaterally infused with vehicle or U0126 in doses of 0.25, 0.5, or 1 µg/hemisphere. One-way ANOVAs revealed significant main effects of E_2_ treatment for OP (*F*_(3,27)_ = 5.12, *p* = 0.006; Fig. 4*A*) and OR (*F*_(3,30)_ = 4.29, *p* = 0.012; Fig. 4*B*). *Post hoc* analyses indicated that the group who received 1 μg/hemisphere of U0126 performed worse than all other groups in both tasks (*p* < 0.02). One-sample *t* tests for OP showed that all groups but the 1-µg group spent significantly more time than chance with the moved object (vehicle, *t*_(9)_ = 2.34, *p* = 0.04; 0.25 µg, *t*_(5)_ = 3.14, *p* = 0.03; 0.5 µg, *t*_(6)_ = 6.41, *p* = 0.0007; 1 µg, *t*_(7)_ = 1.18, *p* = 0.27). For OR, one-sample *t* tests showed that groups infused with vehicle (*t*_(9)_ = 2.66, *p* = 0.03) or 0.5 µg (*t*_(7)_ = 3.86, *p* = 0.006) spent significantly more time than chance with the novel object, whereas the 0.25-µg (*t*_(8)_ = 1.61, *p* = 0.15) and 1-µg (*t*_(6)_ = 1.57, *p* = 0.17) groups did not. It is unclear why the 0.25-µg group would not have shown stronger evidence of learning in OR. Nevertheless, the data suggests that the 0.5 µg/hemisphere dose of U0126 does not affect OP or OR memory consolidation. These results are consistent with our previous findings from OVX mice in which 1.0 μg, but not 0.5 μg, impaired OR and OP memory consolidation (Fernandez et al., 2008; Boulware et al., 2013). Because 0.5 µg was also the highest non-impairing dose of U0126 in the present study, we used the 0.5-µg dose in subsequent experiments to examine the role of ERK phosphorylation in the memory-enhancing effects of E_2_ in sham GDX and GDX males.

Next, sham GDX males were infused with vehicle or 0.5 µg/hemisphere U0126 into the DH and vehicle or 10 µg E_2_ into the dorsal third ventricle immediately after OR and OP training (experiment 4a). DH infusions occurred immediately before intracerebroventricular infusions. The infusion of 10 µg E_2_ intracerebroventricular (same total dose as the two bilateral 5-µg infusions) delivers E_2_ adjacent to the DH while preventing potential tissue damage to the DH from multiple infusions of E_2_ and U0126 in rapid succession (Fortress et al., 2015; Kim et al., 2016). DH-infused U0126 did not prevent intracerebroventricularly-infused E_2_ from enhancing memory consolidation in either task, as indicated by significant main effects of E_2_ treatment for OP (*F*_(1,20)_ = 10.91, *p* = 0.0036; Fig. 4*C*) and OR (*F*_(1,31)_ = 6.91, *p* = 0.0132; Fig. 4*D*) in the absence of significant main effects for U0126 treatment or E_2_ × U0126 interactions. In support of these findings, one-sample *t* tests showed that groups infused with E_2_ + vehicle (OP, *t*_(6)_ = 5.93, *p* = 0.001; OR, *t*_(8)_ = 2.91, *p* = 0.02) or E_2_ + U0126 (OP, *t*_(6)_ = 2.7, *p* = 0.036; OR, *t*_(9)_ = 2.37, *p* = 0.04) spent significantly more time with the moved and novel objects, whereas groups infused with vehicle + vehicle or vehicle + U0126 did not. Similar effects were found in GDX males (experiment 4b). Two-way ANOVAs revealed significant main effects of E_2_ treatment in OP (*F*_(1,27)_ = 20.76, *p* = 0.0001; Fig. 4*C*) and OR (*F*_(1,35)_ = 13.3, *p* = 0.0009; Fig. 4*D*), in the absence of main effects for U0126 treatment or significant interactions. Also consistent with this finding, one-sample *t* tests revealed that GDX males treated with E_2_ + vehicle or E_2_ + U0126 spent significantly more time with the moved object than chance in OP (E_2_ + vehicle: *t*_(7)_ = 4.35, *p* = 0.033; E_2_ + U0126: *t*_(8)_ = 2.61, *p* = 0.03) and with the novel object than chance in OR (E_2_ + vehicle: *t*_(10)_ = 2.8, *p* = 0.02; E_2_ + U0126: *t*_(8)_ = 8.14, *p* = 0.0001). In contrast, males treated with vehicle + vehicle or vehicle + U0126 performed at chance levels in both tasks. These data suggest that ERK phosphorylation is not required for E_2-_ to-enhance OR or spatial memory consolidation.

**Figure 5.**
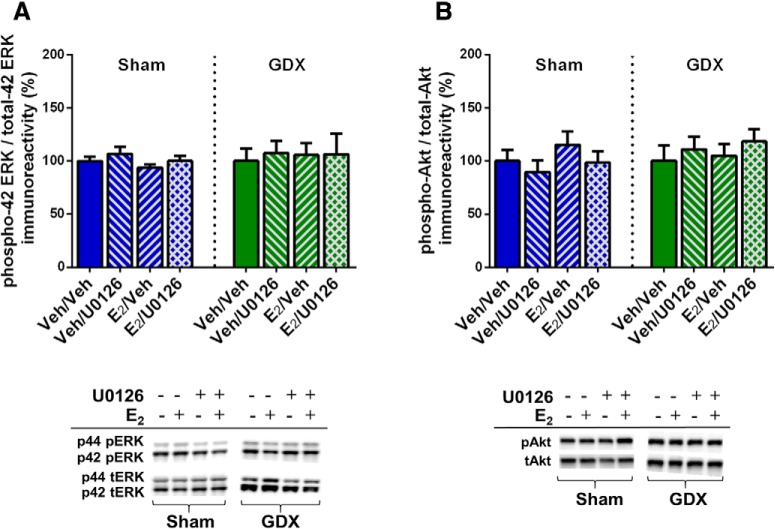
E_2_ did not increase phosphorylated ERK or Akt levels in sham or GDX males, independent of U0126 administration. All groups displayed similar levels of phospho-p42 ERK (***A***) and phospho-Akt (***B***) protein after intracerebroventricular infusion of vehicle or E_2_ and DH infusion of vehicle or U0126. Representative blots for each antibody are shown to below the quantified data. Each bar represents the mean +/− SEM.

To verify that ERK and Akt phosphorylation were not affected by E_2_ or U0126 in the aforementioned behaviorally-tested sham GDX and GDX males, mice were infused two weeks after the conclusion of behavioral testing and DH tissue collected 5 min later. Two-way ANOVAs revealed no significant main effects or interaction between E_2_ and U0126 treatment for phospho-p42 ERK, phospho-p44 ERK, or phospho-Akt in either sham GDX or GDX males (Fig. 5). These data confirm that E_2_ does not increase DH ERK or Akt phosphorylation 5 min after DH infusion in either sham GDX or GDX males. As such, they replicate E_2_’s lack of effect on ERK and Akt in sham GDX males reported in experiment 1 and extend this finding to GDX males, suggesting that testicular hormones do not influence the effects of exogenous E_2_ on these signaling kinases.

Combined, these data demonstrate that ERK phosphorylation in the DH is not required for E_2_ to enhance object recognition and spatial memory consolidation in male mice. These findings are inconsistent with our previous studies in OVX mice in which the same dose of U0126 blocked the memory-enhancing effects of E_2_ on object recognition and spatial memory consolidation (Fernandez et al., 2008; Fan et al., 2010; Fortress et al., 2013), suggesting potentially important sex differences in the signaling mechanisms through which E_2_ regulates memory consolidation.

### E_2_ increases CREB phosphorylation in OVX females, gonadally-intact males, and GDX males

**Figure 6.**
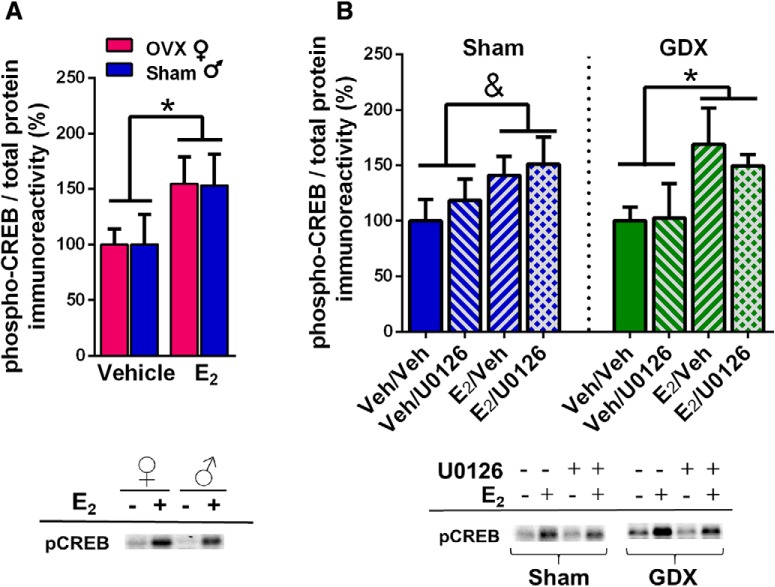
Effects of E_2_ on levels of phospho-CREB in the DH of OVX females, sham males, and GDX males. ***A***, DH infusion of E_2_ significantly increased levels of phospho-CREB in the DH of OVX females and sham males relative to same-sex vehicle-infused mice (*p < 0.05). ***B***, In GDX males, E_2_ significantly increased phospho-CREB levels in the DH (*p < 0.05), and administration of U0126 did not block this increase. In sham males, there was a trend for E_2_ to increase phospho-CREB levels in the DH regardless of U0126 infusion (&*p* = 0.079). Representative blots for each antibody are shown to below the quantified data. Each bar represents the mean +/− SEM.

Given the apparent sex differences in the role ERK phosphorylation in E_2_-induced memory enhancement, we decided to examine possible E_2_ effects on the transcription factor CREB. ERK increases CREB phosphorylation (Adams and Sweatt, 2002), yet E_2_ increases CREB phosphorylation in cultured female, but not male, hippocampi (Boulware et al., 2005). Due to the sex differences in E_2_-induced ERK phosphorylation observed in our studies, we hypothesized that E_2_ would increase CREB phosphorylation in females, but not males. Homogenates from mice in experiments 1 and 4 above were assayed for phospho-CREB levels. We first compared OVX females to intact males (experiment 1). Contrary to our hypothesis, E_2_ increased DH CREB phosphorylation in both sexes (Fig. 6*A*), as suggested by a significant main effect of E_2_ treatment (*F*_(1,20)_ = 5.06, *p* = 0.04) in the absence of a significant main effect of sex or sex × E_2_ treatment interaction. This finding suggested that E_2_ activates CREB in males in a manner independent of ERK phosphorylation. To further support this conclusion, we assayed CREB phosphorylation in homogenates from experiment 4, in which sham GDX and GDX males infused with U0126 or vehicle (DH) and E_2_ or vehicle (intracerebroventricular). Although a two-way ANOVA only reported a trend for an E_2_ enhancement of phospho-CREB in sham mice (*F*_(1,18)_ = 3.46, *p* = 0.079; Fig. 6*B*), there was a significant main effect of E_2_ treatment in the GDX mice (*F*_(1,18)_ = 5.65, *p* = 0.03; Fig. 6*B*). Moreover, no main effects of U0126 treatment or E_2_ × U0126 interactions were significant. Together, these data suggest that E_2_ treatment may enhance memory in male mice through the phosphorylation of CREB in a manner independent from ERK or Akt signaling.

## Discussion

The current study demonstrates a novel sex difference in the molecular mechanisms through which E_2_ enhances memory consolidation. In both sexes, E_2_ infused into the DH enhanced object recognition and spatial memory consolidation in the OR and OP tasks, but the cell-signaling mechanisms employed appear to differ. Our laboratory previously demonstrated that the ability of systemic or dorsal hippocampally-infused E_2_ to enhance memory consolidation in OVX females depends on DH p42-ERK phosphorylation (Fernandez et al., 2008; Fan et al., 2010; Zhao et al., 2010; Fortress et al., 2013). Here, E_2_ infusion did not increase p42-ERK phosphorylation in intact or GDX males, nor was ERK phosphorylation required for E_2_ to enhance memory consolidation in males. Comparison of these data sets strongly suggests that E_2_ regulates memory consolidation via different cell-signaling pathways in males and females, although the signaling mechanisms employed in males remain unclear. Interestingly, CREB phosphorylation was increased by E_2_ in both sexes, and the increase in males was not dependent on ERK phosphorylation, suggesting the involvement of other signaling pathways that activate CREB.

This study is the first to show that acute DH E_2_ infusion can enhance object recognition and spatial memory consolidation in gonadally-intact and GDX males. This finding is consistent with previous reports that chronic systemic E_2_ facilitates memory formation among intact or GDX males tested in various spatial tasks (Luine and Rodriguez, 1994; Heikkinen et al., 2002; Gibbs, 2005; Locklear and Kritzer, 2014), and studies using acute or chronic E_2_ in similar object-based tasks to those used here (McConnell et al., 2012; Jacome et al., 2016). The present study is particularly unique in directly comparing effects of E_2_ in males and females, as well as intact and GDX males, within the same study. Several studies report memory deficits after GDX (Ceccarelli et al., 2001; Kritzer et al., 2001, 2007; Daniel et al., 2003; Sandstrom et al., 2006; Aubele et al., 2008; Gibbs and Johnson, 2008; Spritzer et al., 2008; Locklear and Kritzer, 2014), some of which can be rescued by E_2_ administration (Luine and Rodriguez, 1994; Gibbs, 2005; Gibbs and Johnson, 2008). Although the particular testing delays used in the present study precluded assessment of GDX effects on memory consolidation, the data clearly show that the dorsal hippocampi of both GDX and intact males are responsive to exogenous E_2_, independent of circulating androgens and estrogens. The male hippocampus can aromatize its own E_2_ from androgen precursors (Fargo et al., 2009), so this responsiveness may result from sensitivity to *de novo* E_2_ synthesis within the DH. This study did not directly test a potential role for hippocampal-synthesized E_2_ in the mnemonic responses of males, but preliminary and published data from our lab suggest that aromatase inhibition in the DH blocks OR and OP memory consolidation in GDX female and male, but not intact male, mice (Tuscher et al., 2016b; Koss et al., 2018). Additional work will be necessary to ascertain the relative role of testicular and hippocampal E_2_ to memory consolidation in males.

Although E_2_ enhanced memory in males, the underlying molecular mechanisms appear to differ between the sexes. In OVX mice, E_2_ enhances memory consolidation by sequentially activating PI3K/Akt, ERK, and mTOR signaling in the DH (Fernandez et al., 2008; Fan et al., 2010; Fortress et al., 2013). DH infusion of U0126 or an mTOR inhibitor not only prevents E_2_ from enhancing memory (Fernandez et al., 2008; Fortress et al., 2013), but also from increasing CA1 dendritic spine density (Tuscher et al., 2016a) in OVX mice. As such, rapid ERK activation is essential for effects of E_2_ on both memory consolidation and synaptic morphology in OVX females. In contrast, using the same methods and doses of E_2_ and U0126, we report here that E_2_ did not increase DH ERK phosphorylation, nor did inhibiting ERK activation block E_2_-induced memory consolidation, in intact or GDX males. Although the inability of U0126 to block the memory-enhancing effects of E_2_ could be related to sex differences in interactions between E_2_ and U0126, this unlikely because: (1) our U0126 dose-response data are identical in males (this study) and females (Fernandez et al., 2008; Boulware et al., 2013), and (2) our Western blottings in males repeatedly showed no effect of E_2_ on ERK or Akt phosphorylation in males. Together, our previous findings and the present study indicate that the cell-signaling mechanisms underlying the memory-enhancing effects of DH-infused E_2_ differ between the sexes.

The molecular basis for the discrepant roles of ERK in memory consolidation among females and males may lie in sex differences in estrogen receptor (ER) distribution or function. Although overall hippocampal ERα and ERβ distribution is similar in males and females (Romeo et al., 2005; Mitterling et al., 2010), differences in subcellular ER localization could contribute to the sex differences in cell signaling shown here. For example, E_2_ potentiates hippocampal glutamateric synaptic transmission via different ER mechanisms in GDX male and female rats; glutamate release probability is regulated presynaptically by ERβ in females and ERα in males, whereas glutamate sensitivity is mediated postsynaptically by GPER in females and ERβ in males (Oberlander and Woolley, 2016, 2017). Interestingly, GPER activation in the DH does not phosphorylate ERK or depend on ERK activation to enhance OR and OP memory consolidation in OVX female mice; rather it enhances memory by activating c-Jun N-terminal kinase (JNK; Kim et al., 2016). Thus, sex differences in ER distribution could lead to ERK-independent signaling in males mediated by GPER or other receptors, like glutamatergic receptors. In OVX female mice, metabotropic (mGluR1a) and ionotropic (NMDA) glutamate receptors are necessary for E_2_ to activate ERK and enhance OR and OP memory consolidation (Lewis et al., 2008; Boulware et al., 2013). ERα and ERβ enhance OR and OP memory consolidation in OVX females by interacting with mGluR1a at the membrane to, most likely, potentiate the G-protein signaling that occurs as mGluR1a is bound by glutamate (Boulware et al., 2013). Additionally, E_2_ increases NMDA neurotransmission in female rats by recruiting existing NMDA receptors to synaptic sites (Jelks et al., 2007; Snyder et al., 2011), although it is unknown whether E_2_ affects glutamate receptors similarly in males. E_2_ increases long-term potentiation (LTP) amplitude and reduces LTP threshold in both males and females (Smith and McMahon, 2006; Kramár et al., 2009; Smejkalova and Woolley, 2010; Tanaka and Sokabe, 2013; Kumar et al., 2015), but these effects depend on NMDA receptors in females and AMPA receptors in males (Smith and McMahon, 2006; Kramár et al., 2009). Thus, sex differences in interactions between E_2_ and glutamate receptors could lead to differential cell signaling in males and females.

The activation of CREB in the DH by E_2_ in both sexes is consistent with work in male and female mice showing a similar phosphorylation of CREB in the DH by E_2_ (Abrahám and Herbison, 2005). However, that same study reported sex differences in E_2_-induced CREB activation among hypothalamic and basal forebrain nuclei. Another study using neonatal cultured hippocampal neurons from male and female rats demonstrated that E_2_ activated ERK, CREB, and CRE-mediated gene transcription in females but not males (Boulware et al., 2005). These inconsistencies may be due to differences in species, *in vitro* versus *in vivo* methodologies, or hippocampal subregion specificity. For example, our lab demonstrated that contextual fear conditioning induced greater p42 ERK phosphorylation in the ventral, but not dorsal, hippocampus in male rats relative to females (Gresack et al., 2009). However, the present study and that by Abrahám and Herbison (2005) both report that E_2_ increases CREB phosphorylation *in vivo* in the DH of adult male and female mice, suggesting some degree of consistency in this finding.

CREB phosphorylation is important for hippocampal dendritic spinogenesis, synaptic plasticity, and memory consolidation (Murphy and Segal, 1997; Lonze and Ginty, 2002; Alberini, 2009; Kida, 2012). CREB can be activated through multiple independent cell-signaling pathways including ERK, protein kinases A and C (PKA, PKC), multiple calcium/calmodulin kinases (CaMKs), and p38 MAPK (Lonze and Ginty, 2002). Our data from both sham and GDX males suggest that ERK activation is not necessary for E_2_ to increase pCREB levels in the DH. It is unclear if the same is true for females because our previous studies of E_2_ and U0126 infusion in OVX mice did not measure pCREB levels; however, we doubt this because E_2_-induced alterations in memory consolidation, epigenetic processes, and local protein translation depend on ERK phosphorylation in OVX mice (Zhao et al., 2010; Fortress et al., 2013). Among males, other kinases could mediate the effects of E_2_ on memory and CREB, given the multitude of other signaling molecules that regulate CREB phosphorylation. In studies using cultured embryonic hippocampal neurons, in which sex was not reported, E_2_ increased CREB phosphorylation in a manner dependent on Ca^2+^, NMDA receptors, ERK, PKA, and CaMKII (Murphy and Segal, 1997; Zhao et al., 2005; O'Neill et al., 2008), suggesting perhaps a role for p38 MAPKs or PKC. However, in other embryonic hippocampal cultures, the potent androgen dihydrotestosterone activates CREB through PKC without activation of ERK, CaMKIV, or PKA (Nguyen et al., 2009). Thus, it is possible for hormones to regulate CREB via some kinases and not others. Given the lack of *in vivo* data on this subject, more research in both sexes is clearly needed to parse the mechanisms underlying the memory-enhancing effects of E_2_.

The current study demonstrates novel sex differences in the cell-signaling mechanisms through which E_2_ regulates memory consolidation. The present data are consistent with previous studies showing that E_2_ can enhance memory consolidation in both males and females. However, whereas ERK signaling is necessary for this effect in females, it plays no role in GDX or intact males. Sex differences in molecular means to the same phenotypic end could greatly influence the development of treatments to reduce memory dysfunction conditions such as drug addiction, Alzheimer’s disease, and developmental disorders which differ in severity or risk between the sexes. Sex differences in function at the subcellular level may provide insight into the nature of sex differences in these conditions and lead to more effective, possibly sex-specific, treatments for neuropsychiatric and neurodegenerative diseases.

## Synthesis

Reviewing Editor: Rae Silver, Columbia University Decisions are customarily a result of the Reviewing Editor and the peer reviewers coming together and discussing their recommendations until a consensus is reached. When revisions are invited, a fact-based synthesis statement explaining their decision and outlining what is needed to prepare a revision will be listed below. The following reviewer(s) agreed to reveal their identity: Paul Mermelstein, Jessica Mong. Note: If this manuscript was transferred from JNeurosci and a decision was made to accept the manuscript without peer review, a brief statement to this effect will instead be what is listed below.

Overview:

The manuscript entitled “Sex differences in the rapid cell signaling mechanisms underlying the memory-enhancing effects of 17beta-estradiol” focus on the role of and potential mechanisms used by estradiol (E2) in mediating memory formation in males. While E2's effects on memory consolidation in in females is the focus of a great many studies that have elucidated many potential signaling pathways in the hippocampus, there exists a paucity of like studies in males. Thus, there are significant knowledge gaps in the understanding of E2 mediated action in the male hippocampus. In the present the study, the authors make an important attempt to investigate potential sex differences whether and how E2 mediates dorsal hippocampal (DH) function. The authors report that E2 infused into the DH enhances memory function as previously reported in females. However, in females, where ERK and Akt signaling regulate estradiol enhancement of female memory, these signaling pathways appear nonessential in males. Estradiol does induce CREB phosphorylation in both sexes.

The findings are exciting and novel. However, it is especially important to note that not all experiments in the paper include a direct comparison of male and females (a gap). That said, there are direct comparisons between the sexes that have yielded novel finding. In summary, the work in this manuscript clarifies a sex difference in the effect of estradiol on hippocampal-based memory. Effects of estradiol infusion directly into the hippocampus were indistinguishable between intact and castrated animals.

Major Comments:

Starting with Figure 4, comparisons are made primarily between Sham and GDX males, where no differences are found. An important comparator is males versus females. If possible these comparisons should be added. If the data is not available, the caveat should be very clearly mentioned in the Discussion section of the manuscript and also indicated in the abstract.

In addition, a visual abstract will enhance the readability of the paper.

Additional questions arising:

1. Both the introduction and the discussion lack a clear discussion of E2 aromatization. While the current data suggest this is not a potential mechanism, it is an important concept that should be discussed in more detail. Additionally, this work can not rule out the possibility of de novo E2 synthesis in males. Again the discussion should indicate these possibilities.

2. Figure 4 examines concentrations at which U0126 inhibits memory on its own, then uses a lower concentration to try to block estradiol-mediated improvement of memory. Does the following dose: 0.5ug U0126 block estradiol enhancement of memory in females.

Dose of infused E2. It is not clear if the 5ug per side (and 10ug ICV) is a physiological or pharmacological dose. Was dose response preformed in previous studies for this dose? This may be potentially important in the inhibitor (U0126) as if E2 is at a supraphysiological/pharmacological dose it may mask the inhibitory actions of U0126.

3. The inclusion of figure 5 in the manuscript appears superfluous.

4. In figure 6b: the effect of U0126 on estradiol-induced pCREB might be compared between males and females.

5. In addition, the authors might look to see if inhibitors of PKA and/or CaMK signaling block pCREB. If one of these signaling pathways is identified to block CREB phosphorylation, it should be determined whether it blocks estradiol enhancement of male memory, thus, providing significant insight into the observed sex differences and have a much larger impact in the field.

6. Page 7 lines 135-140: the details of the experimental paradigm for the memory task are not clearly written. The interjection of the references supporting the work in females breaks the clarity of the methodology. Perhaps the authors would consider putting the justifications either in the beginning or at the end after a clear description of the behavioral task. Please clarify the timing of the task. Are the drugs administered 3 hours post training (Line 140)? What is meant by predetermined delay.

7. Page 9 Line 195 and Figure 3 (experiment 3): it is not clear that reporting the western data as % of vehicle is entirely appropriate here. Judging from the representative blots in figure 3b, it appears that the males +/- E2 may have a greater level of pAKT than females without E2. This may represent a potential sex difference, where males have a greater baseline pAKT or alternatively locally derived E2 may be influencing these levels. Nevertheless, the authors should consider running this statistical comparison.
